# Supplementary material for: Understanding disruptions in cancer care to reduce increased cancer burden
Source: eLife. 2023 Aug 10;12:e85024. doi: 10.7554/eLife.85024 (PMC10449381; doi:10.7554/eLife.85024)
Supplement: Supplementary file 1. [file elife-85024-supp1.docx]

Supplemental File 1: Survey item information

| **Construct** | **Survey item and response options** | **Variable coding and interpretation** | **Measure source** |
| --- | --- | --- | --- |
| **Scheduled care and care disruptions** | | | |
| Care delay (primary outcome) | Due to COVID-19, did you decide to: Cancel a scheduled in-person general medical appointment not already cancelled due to COVID-19; Not included, but related items: Cancel a scheduled in-person dental appointment not already cancelled due to COVID-19; Avoid seeking care in a hospital (e.g. labor and delivery, emergency room, etc.)  *Response options: a) yes, b) no, c) does not apply* | Yes for general medical appointment = care disruption  *(Dental and hospital care not included in care disruption primary outcome)* | Adapted from Penedo et al., 2020^30^ |
| Care delay (primary outcome) | Did you or your doctor postpone your mammography because of COVID-19?  *Response options: a) yes (it was me), b) yes (it was my doctor or clinic), c) no, d) don’t know/not sure* | Yes (a or b) = care disruption | Penedo et al., 2020^30^ |
| Care delay (primary outcome) | Did you or your doctor postpone your Pap test because of COVID-19?  *Response options: a) yes (it was me), b) yes (it was my doctor or clinic), c) no, d) don’t know/not sure* | Yes (a or b) = care disruption | Penedo et al., 2020^30^ |
| Care delay (primary outcome) | Did you or your doctor postpone your stool blood test because of COVID-19?  *Response options: a) yes (it was me), b) yes (it was my doctor or clinic), c) no, d) don’t know/not sure* | Yes (a or b) = care disruption | Penedo et al., 2020^30^ |
| Care delay (primary outcome) | Did you or your doctor postpone your colonoscopy because of COVID-19?  *Response options: a) yes (it was me), b) yes (it was my doctor or clinic), c) no, d) don’t know/not sure* | Yes (a or b) = care disruption | Penedo et al., 2020^30^ |
| Care delay (primary outcome) | Did you or your doctor postpone your PSA test because of COVID-19?  *Response options: a) yes (it was me), b) yes (it was my doctor or clinic), c) no, d) don’t know/not sure* | Yes (a or b) = care disruption | Penedo et al., 2020^30^ |
| Cancer Care delay | Were you scheduled for any cancer-related medical care that you or your doctor had to cancel or postpone during the COVID-19 restrictions?  *Response options: a) yes, I had to cancel or postpone, b) yes, my doctor had to cancel or postpone, c) no, d) don’t know/not sure*  **Not included in outcome measure* |  | Penedo et al., 2020^30^ |
| Telehealth appointment | Did you participate in a Telehealth medical appointment (e.g., Zoom, Facetime) since the COVID-19 pandemic?  *Response options: a) yes, b) no* | Yes = participated in telehealth appointment | Adapted from Penedo et al., 2020^30^ |
| Telehealth appointment | *(if yes to previous item)*: Did you participate in a Telehealth appointment for:  *Response options: a) cancer care, b) general health care, c) both* | Response options correlate to telehealth care type | Team created |
| Appointment rescheduling needs | If you were unable to keep a doctor’s appointment for general health care or cancer screening during the COVID-19 pandemic, what do you need most to be able to reschedule that appointment? I need:  *Response options (select one): a) To know my doctor’s office or clinic is taking the appropriate COVID related safety precautions, b) Time to schedule the appointment, c) To know if my doctor’s office is still open, d) To know my doctor’s office is making appointments for general or routine care, e) To know how I’m going to get to the doctor’s office (transportation), f) To know how I’m going to pay for the doctor’s appointment, g)To know that someone can come with me, h) I don’t need anything, i) I am dealing with other things and not ready to reschedule yet, j) Other (please specify)* | Selection of any response a-g, j indicates a need for rescheduling; h or i response considered no need | Adapted from Penedo et al., 2020^30^ |
| **Demographic and social stratification variables** | | | |
| Age | What is your age? | Age measured as a continuous variable in years | Team created |
| Race | Which of these groups would you say best represents your racial identity? (Please mark all that apply)  *Response options: a) American Indian or Alaska Native, b) White, c) Black or African American, d) Asian, e) Middle East and North African (MENA), f) Native Hawaiian or Other Pacific Islander, g) Another group (please specify)* | Combined race and ethnicity recoded into 3 categories: Non-Hispanic Black/African American, Non-Hispanic White, any other race/ethnicity | Adapted from BRFSS 2018^32^ |
| Ethnicity | Are you Hispanic, Latino/a, or Spanish origin? *Response options: a) yes, b) no* |  | Adapted from HINTS 2018^25^ |
| Gender | What is your gender identity?  *Response options: a) Man, b) Woman, c) Transgender, d) Queer/Non-binary, e) Agender/ No gender, f) Something else (please specify), g) Prefer not to answer* | Recoded into 3 categories: a = man, b = woman, c-f = transgender/gender diverse | Adapted from Killermann, 2020^26^ |
| Sex assigned at birth | What is your sex assigned at birth, on your birth certificate? We ask this question to better understand what health screenings are relevant for you.  *Response options: a) Female, b) Male, c) Prefer not to answer* | Sex assigned at birth correlate to response options | Team created |
| Sexual orientation | What is your sexual orientation?  *Response options: a) Asexual, b) Bisexual, c) Gay, d) Lesbian, e) Pansexual, f) Straight or heterosexual, g) Something else (please specify), h) Prefer not to answer* | Recoded into 3 categories: a-e, g = LGBTQIA+, f = straight or heterosexual, h = prefer not to answer | Adapted from Penedo et al., 2020^30^ |
| Education | What is the highest grade or year of school you completed?  *Response options: a) Never attended school or only attended kindergarten, b) Grades 1 through 8 (Elementary), c) Grades 9 through 11 (Some high school), d) Grade 12 or GED (High school graduate), e) Some college, but did not graduate, f) Associate degree or Technical School Certification, g) College 4 years or more (College graduate), h) Graduate or professional school, i) Other (please specify)* | Recoded into 7 categories: a-c = less than high school, d = high school graduate or GED, e = some college, f = associate degree or technical school certificate, g = college graduate, h = graduate or professional school | Adapted from BRFSS 2018^32^ |
| Income | Thinking about members of your family living in this household, what is your combined annual income, meaning the total pre-tax income from all sources earned in the past year?  *Response options: a) $0 to $9,999, b) $10,000 to $14,999, c) $15,000 to $19,999, d) $20,000 to $34,999, e) $35,000 to $49,999, f) $50,000 to $74,999, g) $75,000 to $99,999, h) $100,000 to $199,999, i) $200,000 or more* | Recoded into 9 categories: less than $10,000, $10,000- $14,999, $15,000-$19,999, $20,000-$34,999, $35,000- $49,999, $50,000- $74,999, $75,000- $99,999, and $100,000+. | HINTS 2018^25^ |
| Rurality | What is the zip code where you live?  *Response option: numerical entry* | Zip codes with RUCC code designation of 4 or greater classified as non-metro | Rural-Urban Continuum Codes, 2019^23^ |
| Pre-COVID employment | Which category best describes your occupational status in February 2020 prior to the stay-at-home orders put in place as a result of the COVID-19 pandemic?  *Response option: a) Employed Full-time, b) Employed Part-time, c) Unemployed, d) Homemaker, e) Student, f) Retired, g) Disabled, h) Other (please specify)* | Response options correlate to employment status | Adapted from Penedo et al., 2020^30^ |
| Individual job loss due to COVID-19 | Have you or anyone in your household experienced the following: Laid off job or had to close own business  *Response options: a) Yes (me), b) Yes (person in home), c) No, d) Don’t know/Not sure, e) Prefer not to answer, f) Not applicable* | Yes (a) = job loss | Adapted from Grasso et al., 2020^28^ |
| Health insurance status | Are you currently covered by any of the following types of health insurance or health coverage plan?  *Response options: a) Indian Health Service or Tribal Health Services, b) A plan purchased through a current or former employer or union, c) A plan that you or another family member buys on your own from an insurance company, including Marketplace plans, d) Medicare, for people 65 and older, or people with certain disabilities, e) Medicaid or any kind of government-assistance plan for those with low incomes or a disability, f) TRICARE, VA, or other Military, g) Any other type of health insurance or health coverage plan (please specify), h) I currently do not have health insurance* | Recoded into 5 categories: b or c = private insurance, d = Medicare, e = Medicaid, h = uninsured, a,f,g = other/unknown | BRFSS 2018^32^ coding based on Wadhera et al., (2019, 2020) & Qi et al., 2019^33-35^ |
| Access to a private vehicle | What kind of transportation do you most often use to get to the places you need to go?  *Response options: a) Either my own or someone else’s private car, van, truck or motorcycle, b) Bus, c) Light rail like the Metrolink, d) Call-a-ride, e) Taxi/Uber/Lyft, f) Other (please specify)* | a = access to private vehicle | Adapted from BRFSS 2018^32^ |
| Healthcare discrimination | 7-item scale; hen getting health care, how often has each experience happened to you: a) Treated with less courtesy than other people, b) Treated with less respect than other people, c) Received poorer services than other people, d) Had a doctor or nurse act as if he or she thinks you were not smart, e) Had a doctor or nurse act as if he or she was afraid of you, f) Had a doctor or nurse act as if he or she was better than you, g) Felt like a doctor or nurse was not listening to what you were saying  *Response options: 1) never, 2) once, 3) 2 or 3 times, 4) 4 or more times* | Mean scores calculated across items; higher score indicates more discrimination | Peek et al., 2011^29^ |

*Note*: Affirmative responses to any one of the seven care delay items indicated the presence of care disruption.
