# Supplementary material for: Understanding disruptions in cancer care to reduce increased cancer burden
Source: eLife. 2023 Aug 10;12:e85024. doi: 10.7554/eLife.85024 (PMC10449381; doi:10.7554/eLife.85024)
Supplement: Supplementary file 2. [file elife-85024-supp2.docx]

Supplemental File 2. Characteristics of residents across Missouri and Southern Illinois by race (July-August 2020).

| Variable | Category | Total Sample^1^ (N=680)   – N (%) | Non-Hispanic Black or African American (N=210)  – N (%) | Non-Hispanic White (N=390)   – N (%) |
| --- | --- | --- | --- | --- |
| Care Disruption (primary outcome) | Yes | 304 (44.7%) | 120 (57.1%) | 207 (53.1%) |
|  | No | 376 (55.3%) | 90 (42.9%) | 183 (46.9%) |
| Gender Identity | Woman | 464 (68.2%) | 147 (70.0%) | 274 (70.3%) |
|  | Man | 206 (30.1%) | 60 (28.6%) | 115 (29.5%) |
|  | Transgender / Gender Diverse | 5 (0.7%) | 2 (1.0%) | 1 (0.3%) |
|  | Prefer not to answer | 5 (0.7%) | 1 (0.5%) | 0 (0%) |
| Sex assigned at birth | Female | 472 (69.4%) | 150 (71.4%) | 276 (70.8%) |
|  | Male | 204 (30.0%) | 60 (28.6%) | 114 (29.2%) |
|  | Prefer not to answer | 4 (0.6%) | 0 (0%) | 0 (0%) |
| Sexual Orientation | LGBTQIA+ | 76 (11.2%) | 24 (11.4%) | 34 (8.7%) |
|  | Straight or Heterosexual | 590 (86.8%) | 182 (86.7%) | 353 (90.5%) |
|  | Prefer not to answer | 14 (2.1%) | 4 (1.9%) | 3 (0.8%) |
| Education | Less than High School or GED | 31 (4.6%) | 12 (5.7%) | 9 (2.3%) |
|  | Grade 12 or GED (High school graduate) | 120 (17.7%) | 41 (19.5%) | 65 (16.7%) |
|  | Some college, but did not graduate | 159 (23.4%) | 48 (22.9%) | 97 (24.9%) |
|  | Associates Degree or Technical School Certification | 111 (16.4%) | 46 (21.9%) | 58 (14.9%) |
|  | College 4 years or more (College graduate) | 143 (21.1%) | 32 (15.2%) | 92 (23.6%) |
|  | Graduate or professional school | 115 (16.9%) | 31 (14.8%) | 69 (17.7%) |
| Annual Household Income | $0 to $9,999 | 57 (8.4%) | 26 (12.4%) | 22 (5.6%) |
|  | $10,000 to $14,999 | 53 (7.8%) | 13 (6.2%) | 32 (8.2%) |
|  | $15,000 to $19,999 | 36 (5.3%) | 14 (6.7%) | 16 (4.1%) |
|  | $20,000 to $34,999 | 105 (15.5%) | 43 (20.6%) | 56 (14.4%) |
|  | $35,000 to $49,999 | 110 (16.2%) | 40 (19.1%) | 65 (16.7%) |
|  | $50,000 to $74,999 | 121 (17.9%) | 31 (14.8%) | 69 (17.7%) |
|  | $75,000 to $99,999 | 91 (13.4%) | 17 (8.1%) | 63 (16.2%) |
|  | $100,000 or more | 105 (15.5%) | 25 (12.0%) | 67 (17.2%) |
| Metro or Non-Metro Area (RUCC codes by ZIP Code) | Metro | 493 (72.5%) | 200 (95.2%) | 230 (59.0%) |
|  | Non-Metro | 187 (27.5%) | 10 (4.8%) | 160 (41.0%) |
| Employment (pre-COVID) | Employed Full-time | 321 (47.4%) | 105 (50.2%) | 174 (44.7%) |
|  | Employed Part-time | 72 (10.6%) | 19 (9.1%) | 43 (11.1%) |
|  | Unemployed | 61 (9.0%) | 27 (12.9%) | 29 (7.5%) |
|  | Homemaker | 65 (9.6%) | 14 (6.7%) | 41 (10.5%) |
|  | Student | 4 (0.6%) | 3 (1.4%) | 1 (0.3%) |
|  | Retired | 84 (12.4%) | 25 (12.0%) | 56 (14.4%) |
|  | Disabled | 62 (9.2%) | 15 (7.2%) | 39 (10.0%) |
|  | Self-Employed/Other | 8 (1.2%) | 1 (0.5%) | 6 (1.5%) |
| Insurance | Private | 314 (46.2%) | 90 (42.9%) | 188 (48.2%) |
|  | Medicare/Medicare + | 126 (18.5%) | 31 (14.8%) | 85 (21.8%) |
|  | Medicaid | 120 (17.7%) | 52 (24.8%) | 51 (13.1%) |
|  | Other/Unknown | 22 (3.2%) | 7 (3.3%) | 11 (2.8%) |
|  | Currently do not have insurance | 98 (14.4%) | 30 (14.3%) | 55 (14.1%) |
| Telehealth appointment | Yes | 233 (34.3%) | 69 (32.9%) | 134 (34.4%) |
|  | No | 447 (65.7%) | 141 (67.1%) | 256 (65.6%) |
| Telehealth appointment type | Cancer Care | 6 (2.6%) | 1 (1.5%) | 2 (1.5%) |
|  | General Health Care | 218 (94.0%) | 66 (95.7%) | 127 (95.5%) |
|  | Both | 8 (3.5%) | 2 (2.9%) | 4 (3.0%) |
| Access to Private Vehicle (own or others) | Yes | 611 (89.9%) | 172 (81.9%) | 369 (94.6%) |
|  | No | 69 (10.2%) | 38 (18.1%) | 21 (5.4%) |
| Laid off Job or had to close own business | Yes | 135 (19.9%) | 55 (26.2%) | 58 (14.9%) |
|  | No | 423 (62.2%) | 119 (56.7%) | 260 (66.7%) |
|  | Don’t Know/Not Sure/Prefer Not to Answer | 15 (2.2%) | 5 (2.4%) | 5 (1.3%) |
|  | Not Applicable | 107 (15.7%) | 31 (14.8%) | 67 (17.2%) |
| Variable | | Mean (SD) | | |
| Age | | 46.2 (12.6) | 45.9 (12.2) | 47.5 (12.8) |
| Discrimination^2^ | | 1.8 (0.8) | 1.9 (0.9) | 1.7 (0.8) |

*Statistically significant difference (p<0.05; Chi-square or Fischer’s test for categorical, t-test or Wilcoxon rank sum for continuous)

^Missing values: 1 Hispanic/Latina(a)/Spanish origin; 1 Education; 2 Income; 3 Employment

^1^ Total sample includes all race/ethnicities

^2^Average score of 7 items on a scale of (1) never, (2) once, (3) 2 or 3 times, and (4) 4 times or more; higher scores indicate more discrimination
